# Large Language Model Summarization of Physician-to-Physician Calls for Interhospital Transfer of Patients With ST-Elevation Myocardial Infarction: Observational Study

**DOI:** 10.2196/88834

**Published:** 2026-06-25

**Authors:** Jesse O Wrenn, Madelaine Behrens, Mary S Hershey, Marc Maldaver, John Mitchell, Trevor Thompson, Austin J Triana, Zain M Virk, Yasemin Akdas, Michael R Cauley, Michael J Ward, Ken Monahan

**Affiliations:** 1 Department of Emergency Medicine, Vanderbilt University Medical Center, 2215 Garland Ave, Light Hall Ste 203, Nashville, TN, 37232, United States, 1 6153220160; 2 Department of Biomedical Informatics, Vanderbilt University Medical Center, Nashville, TN, United States; 3 Division of Emergency Medicine, VA Tennessee Valley Healthcare System, Nashville, TN, United States; 4 Department of Internal Medicine, Vanderbilt University Medical Center, Nashville, TN, United States; 5 Office of Community Health and Engagement, Vanderbilt University Medical Center, Nashville, TN, United States; 6 Geriatric Research, Education, and Clinical Center, VA Tennessee Valley Healthcare System, Nashville, TN, United States; 7 Division of Cardiovascular Medicine, Vanderbilt University Medical Center, Nashville, TN, United States

**Keywords:** ST-elevation myocardial infarction, STEMI, patient transfer, large language models, summarization, delivery of health care, artificial intelligence, AI

## Abstract

**Background:**

Interhospital transfer of patients with suspected ST-elevation myocardial infarction (STEMI) requires timely and robust communication. Clinical uptake of potentially useful information from physician-to-physician phone calls authorizing transfer is low at many institutions, at least in part due to relative inaccessibility of call audio and lack of transcripts or summaries. Large language models (LLMs) can transform text into brief, consistently formatted summaries that could be made available in the electronic health record, thus facilitating the timely availability of clinically relevant data to physicians downstream of the transfer call.

**Objective:**

We sought to assess the feasibility of using transcription and LLM summarization to provide written information summarizing transfer calls by adapting the Physician Documentation Quality Instrument (PDQI) to score generated call summaries and evaluate whether LLMs could effectively summarize a curated set of transfer calls.

**Methods:**

STEMI transfer calls for which our institution was the receiving facility were transcribed and summarized by Whisper and ChatGPT (OpenAI), respectively. Each summary was reviewed by 2 of 7 independent physician raters. Summaries were rated using a Likert scale applied to an 8-domain framework adapted from the PDQI. We calculated summary statistics, including means, SDs, and raw and weighted agreement, and produced visual radar plots to demonstrate ratings for each call. We also performed thematic analysis of reviewers’ comments.

**Results:**

We identified 32 calls, of which 1 (3.1%) was excluded for incompleteness. Raw agreement between raters was 62% (153/248), and the mean of the pairwise weighted κ coefficients was 0.19 (SD 0.30; slight agreement). The mean rating of all summaries across all domains was 4.6 of 5 (SD 0.7). The “useful” (mean 4.8/5, SD 0.5) and “consistent” (mean 4.9/5, SD 0.6) domains were the highest rated, and the “thorough” (mean 4.4/5, SD 1.0) and “hallucination free” (mean 4.4/5, SD 0.9) domains were the lowest rated. The mean score for accuracy was 4.6/5 (SD 0.7). Qualitative analysis found that raters penalized the LLM for inferential hallucinations, although these were often clinically accurate, and discrepancies related to calculation of timing of events.

**Conclusions:**

Despite the limitations inherent in a small pilot cohort, this feasibility study suggests that LLMs can generate accurate and pertinent summaries of interhospital transfer calls for patients with STEMI. Interrater agreement was slight, which may suggest inadequate training of raters, unclear definitions, or a limitation of using the PDQI for this task. We identified several important areas for consideration prior to implementation, including thorough assessment of transcription accuracy, prompt engineering to minimize unwanted LLM behavior, and assessment of the impact of incorporating these summaries into clinical care on clinical outcomes.

## Introduction

ST-elevation myocardial infarction (STEMI) is a cardiovascular emergency that requires prompt access to the recommended reperfusion treatment, primary percutaneous coronary intervention (PCI), to achieve optimal outcomes. Approximately 61% of hospitals in the United States lack PCI capabilities [1], requiring transfer of up to 50% of patients with STEMI [2-4]. Shorter time spent at the transferring hospital is significantly associated with shorter door-to-balloon times and improved mortality rates [5]. The quality of communication between referring and receiving institutions has been identified as a major barrier to effective, timely transfer and, thus, may be a potential target for intervention [6].

At Vanderbilt University Medical Center (VUMC) in middle Tennessee, patients are accepted for transfer during phone calls between referring physicians and VUMC cardiologists. Although digitally recorded, phone calls with *accepting* physicians, who make the decision to accept the patient for transfer, are not readily available for review by *receiving* physicians, who ultimately care for the patient on arrival and may differ from the accepting physician. In addition, the *accepting* physician may not have the opportunity to communicate directly with the *receiving* physician, leading to further degradation of information transfer. Thus, a durable near–primary source account of the clinical details surrounding the transfer would assist in adding an element of continuity across the passage of time and transitions to different physicians.

Large language models (LLMs) may offer potential solutions by summarizing transfer calls into brief, consistently formatted, digestible text that could be made available in the electronic health record. There has been extensive study of digital scribes, or summarization of recorded conversations between physicians and patients, but less work in the domain of phone call transcription and summarization [7-9]. A recent study demonstrates successful summarization of phone calls related to emergency department transfer; however, the process included intermediaries that corrected transcription mistakes and performed manual speaker diarization and labeling [10], steps that may not be practically implemented in particularly time-sensitive conditions such as STEMI and may also not be scalable due to personnel and cost requirements. We sought to assess the feasibility of transcription and LLM summarization of STEMI transfer calls without human intervention using the Physician Documentation Quality Instrument (PDQI) [11], adapted subsequently for evaluation of LLM-generated text [12].

## Methods

### Ethical Considerations

This project was approved by our institutional review board with a quality improvement or nonresearch determination (240725). Therefore, individual consent was not required. All identifiable data were stored on encrypted devices within the secure institutional infrastructure. Participants were not compensated for inclusion.

### Data Acquisition

Our institution maintains a log of all patients admitted, transferred, or diagnosed in-house with STEMI. We used this log to identify all patients transferred to VUMC from an outside institution between January 1 and June 30, 2024, for whom there was a clinical concern for STEMI. The recordings of the transfer calls between associated referring physicians and accepting cardiologists were downloaded from an internal audio database (Encore; version 8.6; DVSAnalytics) and reviewed by a single cardiologist (KM) to ensure that they contained both the clinical information upon which the presumptive diagnosis of STEMI was made and the key decision-making discussion that informed the transfer.

### Transcription and Summarization

Each call was then transcribed using the OpenAI Whisper model with English language and large model size preselected, hosted locally within the secure institutional infrastructure. Transcripts were neither preprocessed nor cleaned prior to LLM analysis. Six transcripts were selected at random for assessment of word error rate (WER). Subtle surname misspellings were not counted as errors, and filler words were excluded from analysis. These verbatim transcripts were analyzed using aiChat, VUMC’s HIPAA (Health Insurance Portability and Accountability Act)-compliant GPT-4o–powered LLM. This software was chosen because it is readily available at VUMC and HIPAA compliant. Although prompt engineering [13], the systematic process of drafting clear instructions for an LLM, can optimize performance, this was outside the scope of our project. The following prompt alone, based on prior qualitative work without any additional specific formatting requests, was used to generate each summary [14]:

Please generate the following for the following conversation:

*“Summary*: (provide the best possible summary according to my request)

*Medications administered*: (provide a list of all medications administered thus far)

*Time*: (provide the number of minutes since the chest pain started)

*EKG findings*: (provide the EKG findings related to the STEMI)

*Labs*: (provide any labs that have returned)

*Vitals*: (provide any vitals or information about hemodynamic stability)”

### Quality Measurement

Audio and aiChat summaries were uploaded to REDCap (Research Electronic Data Capture; Vanderbilt University) [15,16] alongside a modified PDQI tool. Modifications to the PDQI included removal of the “up-to-date” and “synthesized” domains, which were deemed not applicable to this project. The adapted PDQI also included a question about whether the summary was “free from hallucination,” defined for the purpose of this study as containing information not verifiable in the call audio. This definition has been used previously for analysis of artificial intelligence (AI) tools [12]. Three transfer calls from December 2022 were used as a pilot dataset to facilitate familiarity with the rating mechanism and identify ambiguities and/or problems with workflow. To measure the quality of transfer call summaries, each case was assigned to 2 of 7 participating second- and third-year internal medicine resident physicians. Raters were directly queried via email regarding their rotation history, which was verified by one author (KM) against the central schedule posted on the medical center’s intranet. They provided the number of cardiac intensive care unit (CICU) rotations and non-CICU cardiology rotations they had completed at that point in their training. The same pair of raters evaluated no more than 2 transfer calls and were blinded to each other’s scores but not to the study hypothesis. Each rater independently listened to the raw transfer call audio and rated the LLM-generated summary on each PDQI domain from 1 (“not at all”) to 5 (“completely”). Raters did not have access to the transcript. Assignments were made to maximally distribute rater pairings. Raters were given an opportunity to leave narrative comments to describe hallucinations, missing information, or other interesting findings.

### Statistical and Qualitative Analysis

We calculated and reported raw interrater reliability and pairwise weighted κ. Interrater agreement was presented graphically as well as using radar charts. In addition, we reported mean and SD values for each measured domain. We reviewed rater comments from each of the measurements and conducted a thematic analysis. We also reviewed comments from a convenience sample of the 5 summaries with the largest difference between the two assigned raters’ scores across all domains and calculated the differences between raters in each domain.

## Results

### Quantitative Results

**Figure 1.**
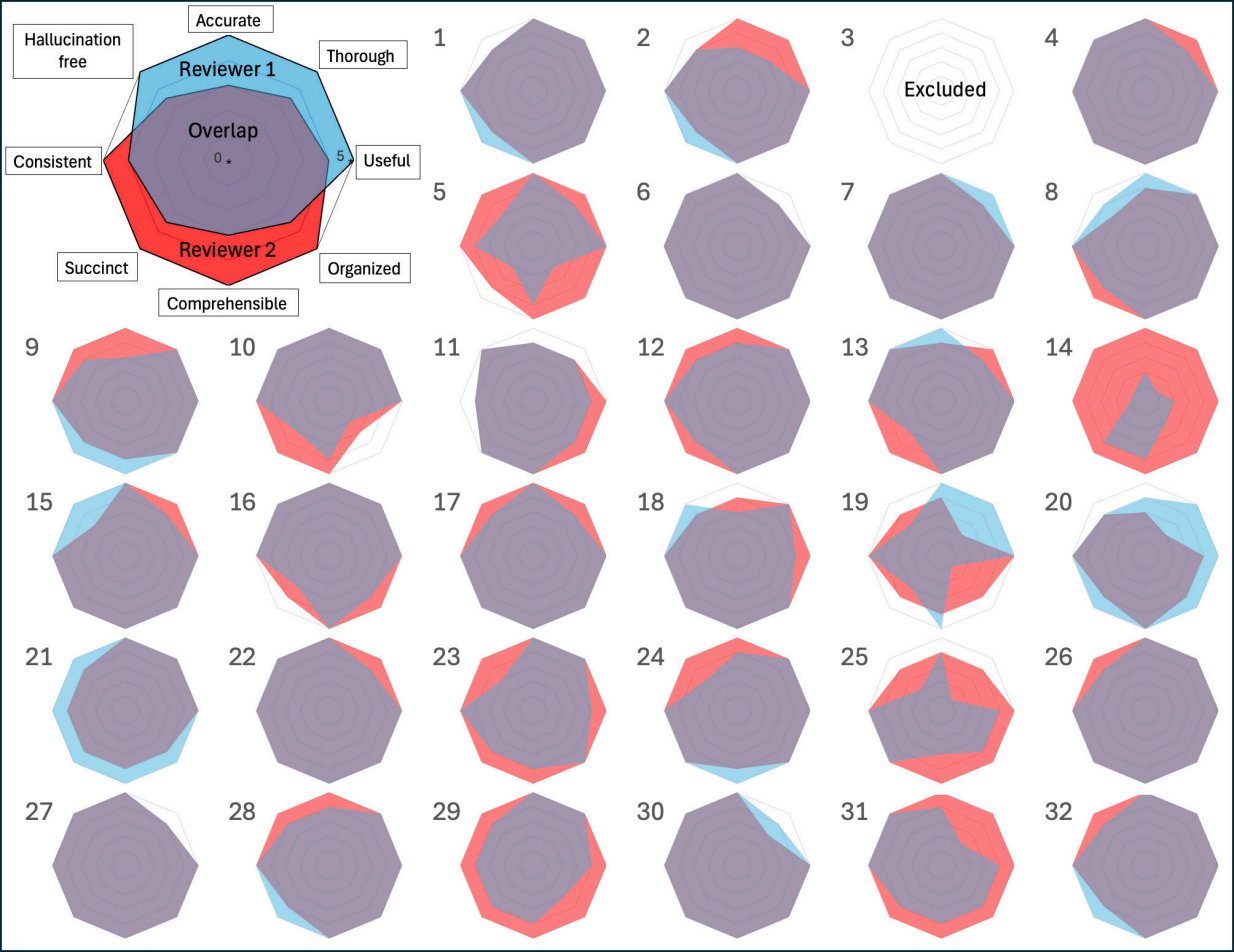
Radar plot representation of interrater variability for each call summary. For a given radar plot, each vertex of the octagon represents one of the adapted Physician Documentation Quality Instrument domains, as depicted in the legend. For each summary, one rater’s scores are shaded in red, and the other rater’s scores are shaded in blue. The overlap between the 2 reviewers is indicated in purple. For example, raters agreed perfectly on the summaries of calls 6 and 27 and disagreed substantially on the summaries of calls 5, 14, 19, 25, and 31.

We identified 32 transfer calls. Of these 32 calls, 1 (3.1%) was excluded from analysis due to the recording being incomplete, leaving a sample size of 31 (96.9%). The average WER for 6 evaluated calls was 1.6% (SD 0.4%), with a 1.4% (70/4865) total WER across the 6 sampled calls. Each call summary was rated on the 8 PDQI domains by 2 raters for a total of 496 ratings or 248 total pairs. At the time of review, the 7 residents rating the summaries had completed an average of 2.4 (SD 1.7) CICU rotations and 3.4 (SD 0.9) non-CICU cardiology rotations. Each rater evaluated 8 or 9 summaries. Of the paired ratings, 62% (153/248) matched exactly between both raters, pairwise weighted κ coefficients ranged from −0.23 (no agreement) to 1 (perfect agreement), and the mean of the pairwise weighted κ coefficients was 0.19 (SD 0.30; slight agreement). Figure 1 shows radar plots of the raters’ scores for each reviewed summary document and displays visually the overall degree of scoring overlap among the rater pairs.

Table 1 provides a summary of ratings by PDQI domain. No summary was rated perfectly by both raters, but there was perfect agreement between raters on summaries 6 and 27. For each of the 8 domains, the mean score exceeded 4 on the 5-point Likert scale. The mean score for all ratings across all domains was 4.6 (SD 0.7).

**Table 1.** Summary statistics by Physician Documentation Quality Instrument domain.

Domain	Score (1-5), mean (SD)
Accurate	4.6 (0.7)
Thorough	4.4 (1.0)
Useful	4.8 (0.5)
Organized	4.6 (0.9)
Comprehensible	4.8 (0.4)
Succinct	4.6 (0.7)
Consistent	4.9 (0.6)
Hallucination free	4.4 (0.9)^a^

a Clinically valid inferences by the large language model were penalized by raters as hallucinations as the inferences were not directly verifiable in the call audio, so the mean score in the “hallucination free” domain should be interpreted with caution.

### Qualitative Results

The descriptive comments by raters identified hallucinations, which often took the form of inferences made by the LLM. For multiple patients, the LLM inferred a patient’s hemodynamic stability from the vital signs provided or from a description of the patient’s appearance as pale or diaphoretic. Although these inferences were not reported as inaccurate, they were not explicitly mentioned in the calls, which prompted the raters to label them as hallucinations. For example, raters noted that the LLM inferred hepatic encephalopathy as a diagnosis (unstated in the call) from the combination of altered mental status and a history of cirrhosis (both stated in the call).

Noninferential inaccuracies were uncommon. However, on several occasions, when the referring physician voiced that a medication was considered or ready to be given, it was cited as given by the LLM. Vital signs were mentioned as misreported once. The raters described more instances of omission of details that they believed would have been useful to the receiving team, including laboratory tests, relevant history, and examination findings. However, raters often praised the summaries for filtering out parts of the conversation that they judged as not important to the receiving team.

Raters identified that the LLM appeared to have difficulty with both absolute and relative time. In total, 6.5% (2/31) of the summaries had events listed in the wrong sequence. Several summaries (7/31, 23%) contained inferences regarding the time of a call that was not explicitly mentioned, both absolute clock time and with respect to the time since the chest pain started, the latter of which was explicitly requested by the prompt.

Interrater agreement calculated using the mean of the pairwise weighted κ coefficients was slight at 0.19 (SD 0.30). A total of 16.1% (5/31) of the summaries reviewed (5, 14, 19, 25, and 31) had notably worse agreement than the others. One specific rater was found to have given the lower average rating in 4 of these 5 summaries and was also a rater on the fifth (summary 19), which had the same average rating between the 2 raters but substantial differences in domain scores. This rater was very thorough with their comments, and most often referred to missing information that the rater believed could alter management, inaccuracies regarding assessment of timing, and inferential hallucinations. In these 5 calls, differences between ratings were the largest in the “thorough,” “hallucination free,” and “organized” domains.

## Discussion

### Principal Findings

#### Overview

This feasibility study explored the use of LLMs to summarize phone calls between referring and accepting physicians in the setting of emergency care for patients with suspected STEMI and evaluated this process using standardized assessment tools. With the caveat that mean scores were derived from ratings with only slight agreement, the high (adapted) PDQI score suggests that the LLM-based approach yielded summaries that were accurate and reproducible. Transfer call summaries, particularly when compared to complete opacity, may represent a transformative solution to address data loss during interhospital transfers. This work demonstrates the feasibility of using an LLM to summarize transcripts of STEMI transfer calls and provides a foundation for further investigation of automated summarization of call audio in patient transfers.

#### Call Summary Quality

No PDQI domain received ratings under 4/5 when averaged over the summaries. The “thorough” and “hallucination free” domains had the lowest scores of the 8 measured domains. Whether a lower score in the “thorough” domain represents the LLM system behaving as designed given inherent limitations of how thorough a summary can be or reflects true information loss is unclear as this was not addressed in the comments left by raters. The distinction is important as redesigning the prompt could generate more thorough, albeit potentially longer summaries. However, the consistently high ratings for the “useful” domain are reassuring and suggest that the lower scores in the “thorough” domain are less likely to be due to diminished transfer of high-yield information.

We found that there were 2 types of hallucinations referred to in the comments regarding the “hallucination free” domain. The first was fabrication, which could clearly cause harm if patients’ management was changed due to inaccurate information. The second was clinical inference (such as inferring hemodynamic instability from words such as “pale” or “diaphoretic”), which was considered reasonable and valid by reviewers but was still labeled as a hallucination because the information was not explicitly verifiable via the call audio. In this initial study, the “hallucination free” rating could represent the worst-case scenario or upper bound, but future work might benefit from parsing further the definition of the hallucination domain by including fabrication and valid inference as 2 separate domains.

Although relatively uncommon, fabrications could potentially adversely influence patient care, particularly if there are no associated primary data included in the summary from which receiving physicians can draw their own conclusions (ie, a receiving physician may interpret vital signs differently from the LLM, but there is no analogous corroborating evidence to support whether a medication was given). Furthermore, if the LLM reported a categorical assessment of a variable such as vital signs and the primary data were not included in the summary, a miscategorized vital sign may prompt misclassification. However, there are strategies for mitigation of LLM hallucinations that were not within the scope of this project [17].

That the LLM had difficulty with the concept of time since the chest pain started is not surprising. Although LLMs have recently achieved significant advances in mathematical reasoning, this remains a challenging frontier in AI research [18]. The prompt we designed requested the number of minutes since the chest pain started, which may require knowledge of the time of the call, the time of onset of chest pain, and the ability to reason mathematically. An alteration of the prompt to request any relevant information from the call on the time when symptoms started as opposed to “the number of minutes since chest pain started” might improve the summary’s accuracy with respect to the timing of relevant events.

There are several additional issues that are important to consider prior to implementation. The average WER was 1.6% (SD 0.4%) for 6 randomly selected transcripts, which is reassuring; however, we did not thoroughly assess the conceptual accuracy of all transcripts, nor did we analyze common errors of transcription. Propagated errors of transcription may have degraded the quality of the summary. Assurance that the transcription model includes medical terminology and drug names is important, as is thorough assessment of transcription quality. Further prompt engineering may reduce the incidence of hallucinations, particularly with respect to the timing of events, including the onset of chest pain. For example, updating the prompt to request only timing that was explicitly discussed in the call may be helpful. Finally, to engender confidence, it may be reasonable to request quotations from the transcript so that physicians can look back to the primary data to confirm critical information.

### Limitations

There were several limitations to this study. The transcription software and LLM were chosen for convenience by the study team, and alternative software or alternate versions could produce different results. The sample size was small, which could limit overall generalizability, although the patient population was relatively homogenous (ie, patients being transferred to a tertiary care center due to concern about STEMI). The selection process included information-rich calls that contained complete clinical and decision-making information, which introduced selection bias. Performance on a randomly selected sample of calls would improve the generalizability of the study to the broader clinical environment, although using an enriched dataset for this feasibility study is a reasonable initial step.

Lack of a comparator such as human expert–generated summaries limits interpretability of the summary quality scores, and the study’s retrospective design limits our ability to understand the clinical adequacy of the summaries and downstream effects of summarization on patient care. A prospective study measuring the impact of incorporation of summaries into clinical workflow on patient outcomes (such as morbidity, mortality, time in the intensive care unit, and overall length of stay) compared to an expert-generated comparator could address some of these design limitations.

Although we did not test different prompts, the prompt used in this study was engineered to optimize the inclusion of key objective data while generating a summary that could be consumed efficiently by a busy clinician. Future work could address this limitation by having receiving physicians evaluate the overall utility of summaries generated using different prompts.

The second- and third-year internal medicine residents reviewing the summaries did not evaluate them from the perspective of interventional or critical care cardiologists; however, they had enough exposure to CICU and general cardiology rotations in their training to reasonably make assessments of summary quality. Furthermore, they are often the intended beneficiaries of call summaries (ie, the first-line recipients of post-PCI patients with STEMI). That said, their relative inexperience could lead to different assessments of the importance of certain elements of the call from those of a more experienced clinician, particularly for the “useful” and “thorough” domains, each of which relies on the communication of information deemed important. Although blinded to the scores of others, raters were not blinded to the LLM-generated source of the summaries, which could introduce rater bias and artificially inflate or deflate scores. They were also not blinded to the study hypothesis, which could introduce confirmation bias as raters could subconsciously inflate scores to align with the goals of the study.

Although infrequent, there were a few substantial disagreements between raters on the quality of summaries, with slight weighted agreement. The reported mean scores for each of the domains must be interpreted with caution given discordant ratings. It is also important to note that the observed slight agreement may partially reflect a statistical ceiling effect as 74.8% (371/496) of the total ratings were 5/5, and κ can underestimate agreement when ratings cluster within a narrow range and chance agreement is inflated. It is unclear whether the remaining disagreement represents inadequate training of raters, unclear definitions, or a limitation of using the PDQI for this task. That one particular rater often provided lower ratings than their counterparts may reflect lenience on the part of the other raters, particularly in light of the possibility of confirmation bias resulting from the fact that raters were unblinded to the study hypothesis. Prior to deployment in clinical practice, in addition to larger transfer call sample sizes and proportionally larger numbers of summary raters, follow-up studies could include additional raters per summary to more robustly characterize the frequency and magnitude of outliers in the domain of interrater disparities.

The original 9-item PDQI (PDQI-9) was developed and validated for the evaluation of notes across 9 criteria. At the conception of this study, we identified an unvalidated adaptation of the PDQI-9 that assessed the quality of LLM-generated text. Since then, there has been a validated evaluation of similar adaptations of the PDQI-9 for LLM-generated text that include the addition of the “hallucination free” domain and the removal of the “up-to-date” domain [19]. There has also been validation of a similar adapted PDQI-9 [20] for LLM-generated summaries that removed the “up-to-date” and “consistent” domains and included the assessment of hallucination in the “accuracy” domain. Notably, the latter defined hallucinations as falsifications (information or data distorted from the original note) or fabrications (made-up information or data that could be plausible but based on nonexistent facts) present in the summary. Regardless, the use of an unvalidated altered instrument may undermine the reliability of the scores to measure true summary quality.

To make this type of automated call summary tool usable in day-to-day clinical practice, the individual components (ie, call transcription and LLM analysis) need to be integrated such that summary generation is transparent to the summary recipient. In addition, the summary would need to be uploaded to the patient’s chart seamlessly, ideally without any incremental effort from the accepting or receiving clinician. Although implementation would require IT support, the use of ambient AI to document outpatient clinic visits [21,22] suggests that this objective is readily achievable in the tertiary medical center environment.

### Conclusions

In a feasibility study, we demonstrated that transcription and LLM summarization can provide information about the content of STEMI transfer calls between institutions and, thus, leveraged primary data that are routinely collected but not optimally used. The use of an adapted PDQI has notable limitations that must be addressed to more accurately assess summary quality, and mean scores must be interpreted cautiously given slight interrater agreement; however, on a subset of curated information-rich transfer calls, summaries were demonstrated to be useful and accurate. Given the risks associated with physician handoffs [23], our results emphasize LLMs’ potential as a transformative tool to address data loss during interhospital transfers, particularly when compared to the current alternative of total information loss.

## Abbreviations

AI: artificial intelligence
CICU: cardiac intensive care unit
HIPAA: Health Insurance Portability and Accountability Act
LLM: large language model
PCI: percutaneous coronary intervention
PDQI: Physician Documentation Quality Instrument
PDQI-9: 9-item Physician Documentation Quality Instrument
REDCap: Research Electronic Data Capture
STEMI: ST-elevation myocardial infarction
VUMC: Vanderbilt University Medical Center
WER: word error rate
